# Clinical Lectures on Diseases of the Eye

**Published:** 1866-10

**Authors:** E. L. Holmes

**Affiliations:** Lecturer on Diseases of the Eye in Rush Medical College, and Surgeon to the Chicago Charitable Eye and Ear Infirmary


### CHICAGO MEDICAL JOURNAL.

Vol. XXIII. OCTOBER, 1866. No. 10.

### ORIGINAL CONTRIBUTIONS.

*Clinical Lectures on Diseases of the Eye.* By E. L. Holmes, M. D., Lecturer on Diseases of the Eye in Rush Medical College, and Surgeon to the Chicago Charitable Eye and Ear Infirmary.

### THE MUSCLES OF THE EYE AND THEIR DISEASES.

Gentlemen,—The presence at the clinic to-day of a case of strabismus and of paralysis of the muscles supplied by the oculomotor nerves, following diphtheria, will be sufficient reason for taking in review what we have said from time to time regarding other similar cases and for extending our investigations of the diseases of the muscles.

It is scarcely necessary to dwell upon the general description of the muscles, for you have seen them demonstrated repeatedly in the dissecting room.

There are, however, a few points which are of particular importance, and yet are scarcely noticed in our text-books.

In commencing the study of this subject, you should be careful to fix in your minds the *exact position of the muscles in the orbit.* You can then more readily perceive the direction in which, by their contractions, their power is applied, and consequently the effect which each muscle exerts upon the globe.

On examining the orbits, you will observe that they are two pyramidal-shaped cavities, situated in such a way, that the sides next to the nasal passages are parallel with each other. The sides next to the temples diverge from each other from behind forward.

The optic foramen, through which passes the optic nerve, and around which the four recti muscles, the superior oblique and the levator of the upper lid, have their origin, is at the posterior portion of each orbit and *near its nasal side.*

The internal rectus lies parallel with the nasal wall of the orbit and parallel with the internal rectus of the other side.

The external rectus, instead of passing directly forward from its origin, passes forward and slightly outward. The superior as well as the inferior rectus on each side passes forward from its origin and slightly *outward.*

With the eye looking directly forward, an imaginary line passed through the centre of the cornea and extended through the centre of the globe posteriorly, which line nearly corresponds to the visual axis, does not fall upon a central point between the origin of the superior and that of the inferior rectus but a little external to it.

This is an important point to bear in mind in studying the action of these two muscles.

The insertion of the four recti muscles in the sclerotic is from two and a half to three lines behind the periphery of the cornea. The insertion of the inferior rectus, according to Arlt, is a trifle nearer that of the internal than of the external recti. Or, in other words, the space between the inferior and external recti is less than between the inferior and external recti.

It may be a matter of interest to know, that these points have been most carefully studied by numerous dissections of the contents of *the orbit in a frozen state.*

Another important point is the fact, that the globes, in their normal state, rotate *in every direction upon their centres.* They *rotate,* and do not, as was once supposed, move bodily on the end of the optic nerve, as the head moves from side to side, or forward and backward on the neck.

As we are told in our anatomies, the superior and inferior recti rotate the cornea upward and downward respectively; the internal and external rotate it inward or outward. A combination of either muscle of each of these pairs, acting with either muscle of the other pair, will turn the cornea obliquely either downward to the right or left, or upward to the right or left.

For distinct (single) vision with two eyes, it is necessary that both eyes move in the same direction, at the same time, and to the same extent.

To accomplish this, in looking downward with both eyes, the two inferior recti act; in looking upward, the two superior recti;—in looking to the left, the external rectus of the left eye and the internal rectus of the right eye, and *vice versa* in looking to the right.

It is one of the functions of the recti muscles, therefore, to direct the visual axes in any desired direction. These muscles are fully competent to accomplish this. But for distinct (single) vision with two eyes, one thing more is absolutely necessary, and that is, that, in all cases, corresponding portions of the two retinas retain their relative position.

I think you can the better comprehend this subject by imagining a line drawn horizontally across the cornea and extended through the globe, dividing it in two hemispheres—the upper and lower. This imaginary line may be called the horizontal great circle of the retina. If the eyes be fixed upon an object directly in front of them, and upon the same level with the centres of the pupils, the image of this object will be formed on the posterior part of the retina and on this horizontal great circle in each eye. Now if the tip of the finger be pressed upon the lower lid of one eye in such a way that the globe is rotated slightly downward, the anterior half of this circle will be depressed, while the posterior portion will be elevated. The image on the eye, thus rotated, will fall upon a point of the retina below this circle. You can therefore see that the image in each eye is on a point of the retina not corresponding with that of the other eye. The result of this is double vision, the two images appearing one above the other. Hence you see that when the visual axis of one eye is directed downwards, it is necessary that the other should also be depressed to the same extent. The same is true of movements in every other direction.

If you will now suppose a line drawn *perpendicularly* through the centre of the cornea and extended through the globe, you divide the eye into two lateral hemispheres. This line is called the meridian of the globe or retina.

Single vision with two eyes requires that the meridians of the eyes should, in all cases, be parallel. If one is inclined to either side, the other must be inclined to the same extent and in the same direction. If the meridian of one should remain perpendicular and that of the other inclined, the image in the latter eye would be thrown on a portion of the retina not corresponding to that receiving the image on the other eye.

To maintain the meridians of both eyes parallel, there is required another pair of muscles—the oblique. The *direction* in which these muscles exert their force is from the posterior and outer toward the front and inner part of the orbit. The *effect* which these muscles will have on the globe will differ according as the direction differs, toward which the pupil (visual axis) is turned.

The attachments of these muscles are so far back on the sclerotic, that the general effect of the superior oblique is to direct the pupil downward and outward, and at the same time to incline the meridian of the globe toward the internal angle; of the inferior oblique, to turn the pupil upward and outward, and at the same time incline the meridian toward the external angle. When, however, the pupil is turned toward the external angle of the eye by the action of the external rectus, the oblique muscles have most influence in inclining the meridian and least influence in elevating or depressing the pupil, (visual axis.) On the other hand, when the pupil is turned toward the internal angle by the action of the internal rectus, the oblique muscles exert most influence in elevating or depressing the pupil and least in inclining the meridian.

This difference in the action of the oblique muscles will be better comprehended by recollecting that when the eye is directed inward, the visual axis is nearer in a line with the direction of the oblique muscles; hence, the superior can exert most influence in depressing and the inferior most influence in elevating this axis.

But when the visual axis is directed outward, it is brought *across* the line in which the oblique muscles lie; hence, these muscles must act principally in rotating the globe on its visual axis.

Since the direction of the superior and inferior recti is forward and outward from their origin, it is evident that, when the pupil (visual axis) is turned to the external angle, these muscles will expend nearly all their force in *elevating* and *depressing* the pupil respectively. When, however, the pupil is turned towards the internal angle, they not only elevate and depress the pupil, but also tend to incline the meridian of the globe inward and outward respectively. This arises from the fact, that in the former case the visual axis is brought almost in an exact line with the course of these muscles; consequently, these muscles, with the globe in this position, have most influence in raising and depressing this axis. In the latter case, the visual axis is so turned as to lie across the line of direction of these muscles. In this position of the globe, the contraction of the inferior and superior recti muscle must tend also to incline the meridian outward or inward respectively, as well as elevate and depress the pupil, (visual angle.)

Now, with these explanations, I think you can understand that, while one eye is turned, for instance, inward and upward, its meridian is slightly inclined, through the action of the superior rectus, inward, and that while the other eye necessarily assumes a position with the pupil turned upward and outward, its meridian must be slightly inclined outward, to maintain its parallelism with the meridian of the other eye. This is accomplished by the inferior oblique slightly drawing the lower portion of the meridian toward the inner angle, thereby throwing its upper portion outward.

In looking downward and inward with one eye, the inferior rectus inclines the meridian outward, by drawing its lower portion toward the internal angle of the eye; at the same time one eye is turned in this direction, the other eye is turned downward and outward. To maintain the meridians of the two eyes parallel, the superior oblique of the latter eye inclines the meridian toward the inner angle by drawing the upper portion of the meridian inward.

The action of the oblique muscles is incorrectly explained in almost every modern work on anatomy and physiology in the English language. It was formerly erroneously supposed that they served to keep the "meridians" of the eye perpendicular when the head was inclined from one shoulder to the other. Their true function is now known to be in connection with the other muscles, especially the inferior and superior recti muscles, *to keep the meridians* PARALLEL, *thereby maintaining corresponding portions of the two retinæ in their relative positions.*

It is still impossible, however, to state precisely what influence each muscle exerts in any particular motion of the eyes. Undoubtedly somewhat different combinations of muscles are employed by different individuals in looking in the same direction, just as different individuals employ different combinations of muscles in certain movements of the fingers and hands, as in playing musical instruments and in performing surgical operations. Hence the differences in gracefulness and pleasant expression in the motions of the eyes in different individuals.

Although certain passive expressions are influenced by the form of the eyes, the color of the iris, shape of the brows and cheeks and other features, the active emotions, such as anger, mental pain, and diffidence, are exhibited in the eyes by their peculiar movements.

In accommodating the eyes for near objects, the two internal recti muscles contract together to produce a greater degree of convergency in the visual axes. These muscles act, therefore, consensually with the ciliary muscle and the annular fibres of the iris in producing a greater degree of convexity of the crystalline lens and in contracting the pupil.

The muscles of the eye, like the muscles of the body, always possess a certain degree of tension upon their antagonists. The rotation of the globe is not produced by a contraction of one set of muscles and a *total relaxation* of the antagonists; but, as one set contracts the other *yields*, without losing its tonicity. The muscles might be compared to the reins in the hands of a skilful driver guiding a spirited horse in a crowded street. Both reins are held firm, although each in turn has slightly the preponderance, as the horse is directed to one side of the street or the other.

The arteries of the muscles of the eye are branches of the ophthalmic artery.

The veins are branches of the ophthalmic and facial vein. These vessels are of almost no surgical importance.

